# The corepressor NCOR1 regulates the survival of single-positive thymocytes

**DOI:** 10.1038/s41598-017-15918-0

**Published:** 2017-11-21

**Authors:** Lena Müller, Daniela Hainberger, Valentina Stolz, Patricia Hamminger, Hammad Hassan, Teresa Preglej, Nicole Boucheron, Shinya Sakaguchi, G. Jan Wiegers, Andreas Villunger, Johan Auwerx, Wilfried Ellmeier

**Affiliations:** 1 Division of Immunobiology, Institute of Immunology, Center for Pathophysiology, Infectiology and Immunology, Medical University of Vienna, 1090 Vienna, Austria; 2 Innsbruck Medical University, Biocenter, Division of Developmental Immunology, Innsbruck, Austria; 3 Ecole Polytechnique Fédérale de Lausanne, Laboratory of Integrative and Systems Physiology, Lausanne, Switzerland; 4 Department of Biochemistry (Shankar Campus), Abdul Wali Khan University (AWKUM) Mardan, KPK, Pakistan

## Abstract

Nuclear receptor corepressor 1 (NCOR1) is a transcriptional regulator bridging repressive chromatin modifying enzymes with transcription factors. NCOR1 regulates many biological processes, however its role in T cells is not known. Here we show that *Cd4*-Cre-mediated deletion of NCOR1 (NCOR1 cKO^Cd4^) resulted in a reduction of peripheral T cell numbers due to a decrease in single-positive (SP) thymocytes. In contrast, double-positive (DP) thymocyte numbers were not affected in the absence of NCOR1. The reduction in SP cells was due to diminished survival of NCOR1-null postselection TCRβ^hi^CD69^+^ and mature TCRβ^hi^CD69^−^ thymocytes. NCOR1-null thymocytes expressed elevated levels of the pro-apoptotic factor BIM and showed a higher fraction of cleaved caspase 3-positive cells upon TCR stimulation *ex vivo*. However, staphylococcal enterotoxin B (SEB)-mediated deletion of Vβ8^+^ CD4SP thymocytes was normal, suggesting that negative selection is not altered in the absence of NCOR1. Finally, transgenic expression of the pro-survival protein BCL2 restored the population of CD69^+^ thymocytes in NCOR1 cKO^Cd4^ mice to a similar percentage as observed in WT mice. Together, these data identify NCOR1 as a crucial regulator of the survival of SP thymocytes and revealed that NCOR1 is essential for the proper generation of the peripheral T cell pool.

## Introduction

Cell fate decisions and lineage specifications during T cell development are accompanied with the establishment and maintenance of cell lineage-specific expression patterns^1^. Lineage-specific genes are induced, while lineage-inappropriate genes are silenced. Epigenetic mechanisms such as DNA methylation and histone modifications regulate the chromatin accessibility for the transcriptional machinery at target genes and play a crucial role in these processes. Chromatin modifying enzymes that regulate reversible changes in histone acetylation or methylation are recruited to gene-specific transcription factors as part of larger multiprotein complexes. This leads to either transcriptional activation or repression of target genes. The outcome depends on the cellular context, the type of the recruiting transcription factor, the composition of the multiprotein complexes and the associated chromatin modifying enzymes^2^.

One important group of transcriptional regulators that bridge repressive chromatin modifying enzymes with specific transcription factors is formed by nuclear receptor corepressor 1 (NCOR1) and its related factor silencing mediator of retinoid and thyroid receptor (SMRT or NCOR2)^3^. NCOR1 was identified as a non-DNA binding corepressor of the transcription factors thyroid hormone receptors and retinoic acid receptors and is essential for mediating transcriptional repression of nuclear receptors in the absence of their ligands^4^, however NCOR1 interacts also with many other types of transcription factors^5^. The repressive activity of NCOR1-containing complexes is mediated via the recruitment of histone deacetylases (HDACs), in particular HDAC3, although association with other HDAC members such as HDAC1, 4, 5 and 7 has been shown as well^2,6^. NCOR1 has been implicated in many biological processes including development, differentiation, cell homeostasis and metabolism^3^. Germline deletion of NCOR1 results in embryonic lethality at E15.5 due to defects in central nervous system development and in definitive erythropoiesis^7^. Conditional gene targeting approaches as well as the generation of transgenic mouse models expressing mutant versions of NCOR1 revealed cell type- and tissue-specific functions for NCOR1 in muscle cells^8^, adipocytes^9^, macrophages^10^ and in the liver^11^.

The role of NCOR1 in T cells is not known. It has been shown in E14.5 *Ncor1* ^−/−^ fetal thymic organ cultures that loss of NCOR1 leads to a severe block at the double-negative (DN) 3 stage with almost no double-positive (DP) cells after 3 days of culture^7^. Moreover, NCOR1 interacts with several members of the BTB domain-containing zinc finger (BTB-ZF) transcription factor family such as PLZF^12,13^, BCL6^13,14^ and MAZR^15^, which itself are crucial regulators of T cell development and function^16,17^. In thymocytes, deletion of HDAC3, the main HDAC family member recruited by NCOR1 to target genes^2^, leads to severely reduced numbers of mature thymocytes and peripheral T cells due to defects at the DN4 to DP transition, during positive selection and post-thymic T cell maturation, dependent on the developmental stage of *Hdac3* deletion^18–21^. Together, these data suggest important functions for NCOR1 in T cells.

Here we investigated the role of NCOR1 during T cell development by using conditional gene targeting approaches. We show that *Cd4*-Cre-mediated T cell-specific loss of NCOR1 (abbreviated *Ncor1* ^f/f^ *Cd4*-Cre and designated as NCOR1 cKO^Cd4^) led to reduced numbers of CD4 and CD8 single-positive (SP) cells, while the number of CD4^+^CD8^+^ DP cells was not affected in the absence of NCOR1. The drop in SP thymocyte numbers in NCOR1 cKO^Cd4^ mice, which was caused by cell-intrinsic effects, resulted also in reduced numbers of peripheral T cells. SP thymocytes were reduced due to impaired survival of positively selected NCOR1-null TCRβ^hi^CD69^+/−^ thymocytes. Further, NCOR1-null thymocytes expressed elevated levels of the pro-apoptotic factor BIM and, upon *ex vivo* anti-CD3/anti-CD28 stimulation, displayed a higher fraction of cleaved caspase 3-positive cells. However, negative selection was not affected, since staphylococcal enterotoxin B (SEB)-mediated depletion of Vβ8-expressing CD4SP thymocytes was not altered. The mature SP cells that developed in the absence of NCOR1 expressed elevated levels of IL-7Rα (CD127) and BCL2, which both promote the survival of positively selected cells. This suggests compensatory mechanisms within the NCOR1 cKO^Cd4^ SP population to overcome elevated expression levels of BIM. Finally, transgenic expression of BCL2 restored the population of TCRβ^hi^CD69^+/−^ thymocytes and hence the development of SP cells to a similar percentage as observed in WT mice. Together, these data identify NCOR1 as a crucial regulator of the survival of SP thymocytes and revealed that NCOR1 is essential for the proper generation of the peripheral T cell pool.

## Results

### Conditional deletion of NCOR1 in T cells leads to reduced numbers of peripheral T cells

**Figure 1:**
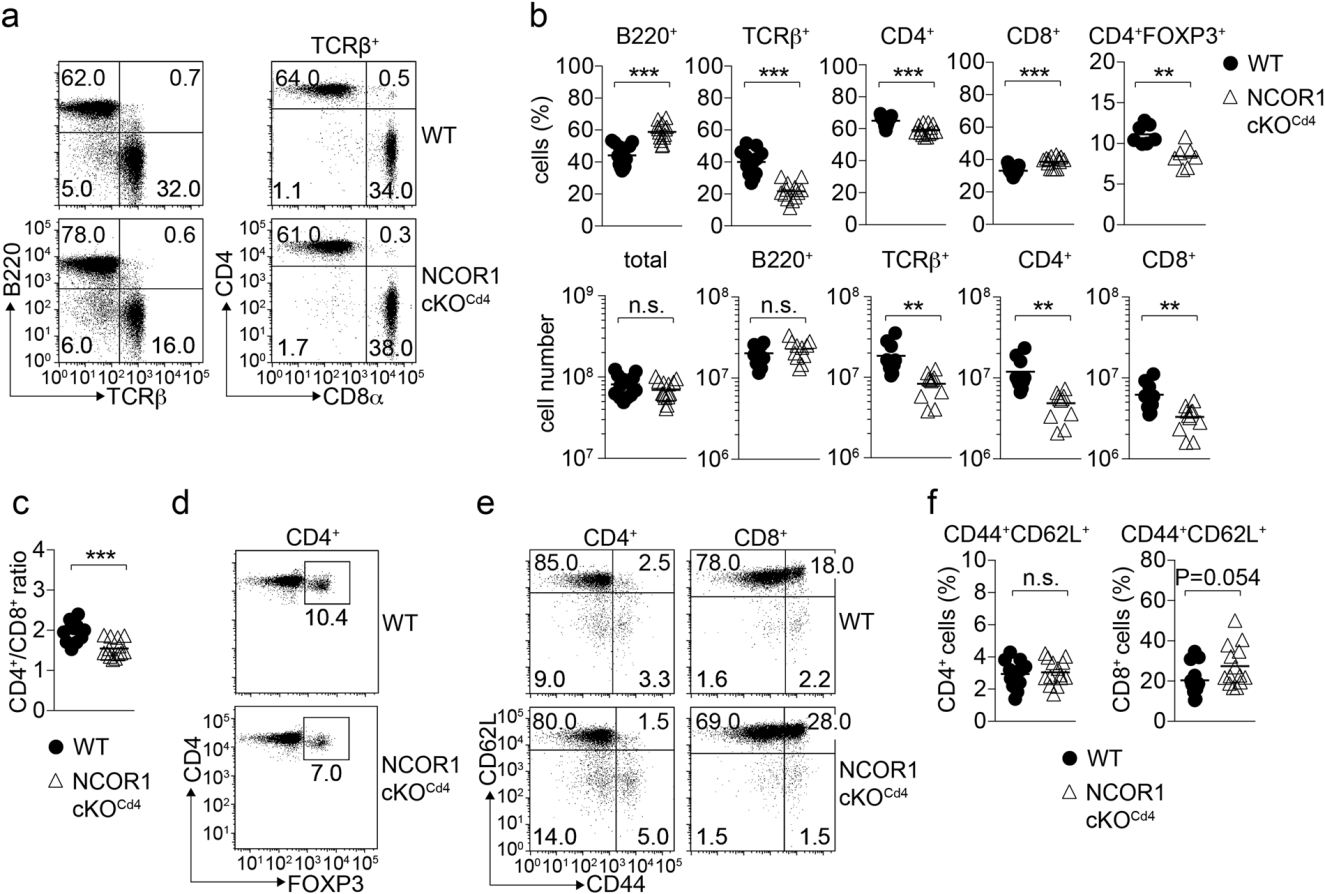
Reduced numbers of peripheral T cells in the absence of NCOR1. (**a**) Flow cytometry analysis of B220, TCRβ, CD4 and CD8α expression on splenocytes isolated from *Ncor1* ^f/f^ (WT) and *Ncor1* ^f/f^ *Cd4-*Cre (NCOR1 cKO^Cd4^) mice. For CD4 and CD8α expression, cells were pre-gated on TCRβ^+^ T cells. (**b**) Percentages and numbers of total cells, B220^+^ cells, TCRβ^+^ cells, CD4^+^ T cells, CD8^+^ T cells and CD4^+^FOXP3^+^ T cells (only percentages) in the spleens of WT and NCOR1 cKO^Cd4^ mice are shown. (**c**) Diagram indicates CD4 to CD8 T cell ratio. (**d**) Flow cytometry analysis of FOXP3 expression on CD4^+^ T cells isolated from the spleen of WT and NCOR1 cKO^Cd4^ mice (pre-gated on TCRβ^+^CD4^+^ T cells). (**e**) Flow cytometry analysis of CD44 and CD62L expression on CD4^+^ and CD8^+^ T cells isolated from the spleen of WT and NCOR1 cKO^Cd4^ mice. (**f**) Percentage of CD44^hi^CD62L^+^ CD4^+^ and CD8^+^ splenic T cells in WT and NCOR1 cKO^Cd4^ mice. (**a**,**d**,**e**) Numbers indicate the percentage of cells in the respective quadrants. (**b**,**c**,**f**) Thick horizontal bars indicate the mean. *P < 0.05, **P < 0.01 and ***P < 0.001 (unpaired two-tailed Student’s t-test). Data are representative (**a**,**d**,**e**) or show the summary (**b**,**c**,**f**) of at least 10 mice (**a**,**b**), 7 mice (**d**) or 13 mice (**c**,**e**,**f**) that were analyzed in at least 3 (**a**,**b**,**d**) and 4 (**c**,**e**,**f**) independent experiments.

To reveal the function of NCOR1 in the T cell lineage, we generated mice with a T cell-specific deletion of *Ncor1*. Since it has been reported that NCOR1-null fetal thymocytes have a block at the DN stage during T cell development, we crossed mice carrying a conditional “floxed” *Ncor1* allele (*Ncor* *1* ^f/f^)^8^ with the *Cd4*-Cre deleter strain^22^ to generate *Ncor* *1* ^f/f^ (WT) and *Ncor* *1* ^f/f^ *Cd4-*Cre (NCOR1 cKO^Cd4^) mice. In comparison to WT mice, NCOR1 cKO^Cd4^ mice displayed a 2-fold reduction of the percentage and number of peripheral T cells (Fig. 1a,b). Within the TCRβ^+^ subset in NCOR1 cKO^Cd4^ mice, the frequencies of CD8^+^ T cells and CD4^+^ T cells were slightly increased and decreased, respectively, (Fig. 1a,b upper panel), leading to a mild change in the CD4/CD8 ratio in the absence of NCOR1 (Fig. 1c). Further, there was a relative reduction of FOXP3^+^ regulatory T cells within the already reduced CD4^+^ T cell population (Fig. 1b and d). The CD44^hi^CD62L^+^ subset within the CD8^+^ T cell, but not within the CD4^+^ T cell population, was slightly enhanced in NCOR1 cKO^Cd4^ mice (Fig. 1e,f). Peripheral CD4^+^ and CD8^+^ T cells did not have a remaining *Ncor1* allele, indicating that no T cells escaped the deletion of *Ncor1* in NCOR1 cKO^Cd4^ mice (Supplementary Figs S1a and S2a).

### Loss of NCOR1 leads to reduced numbers of single-positive thymocytes due to a T cell-intrinsic effect

**Figure 2:**
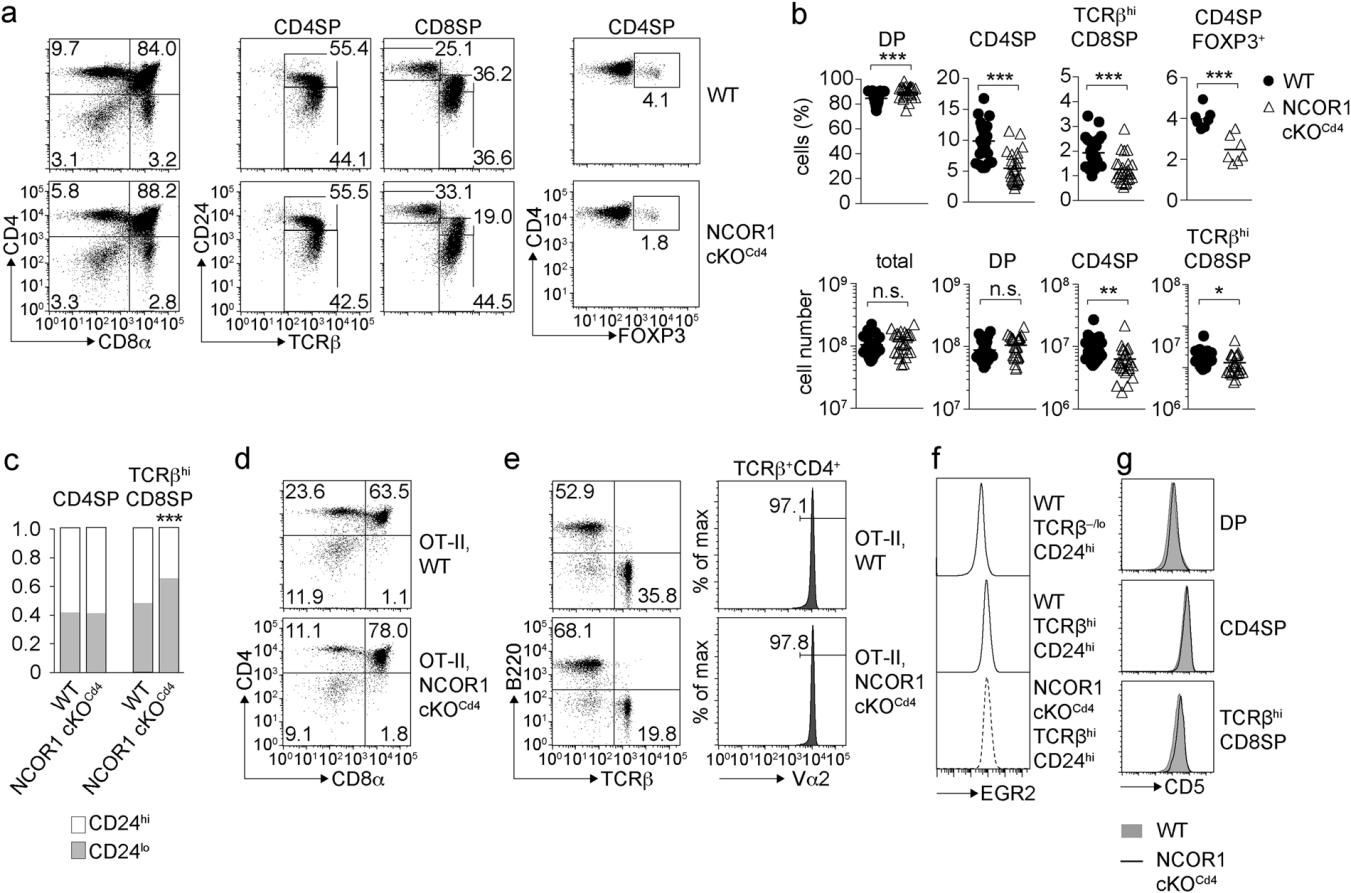
Mature SP thymocytes are reduced in NCOR1 cKO^Cd4^ mice. (**a**) Flow cytometry analysis of CD4, CD8α, TCRβ, CD24 and FOXP3 expression in WT and NCOR1 cKO^Cd4^ thymocytes. The middle panel shows CD24 and TCRβ expression on CD4SP and CD8SP thymocytes. (**b**) Percentages and cell numbers of all thymocytes, DP, CD4SP and TCRβ^hi^ CD8SP thymocytes, as well as percentages of FOXP3^+^ T cells within the CD4SP subset in WT and NCOR1 cKO^Cd4^ mice. (**c**) Bar diagrams show the relative distribution of CD24^hi^ and CD24^lo^ cells within the CD4SP and TCRβ^hi^ CD8SP subsets. (**d**) Expression of CD4 and CD8α on thymocytes isolated from OT-II,WT and OT-II,NCOR1 cKO^Cd4^ littermates. (**e**) Flow cytometry analysis of B220 and TCRβ expression on splenocytes isolated from OT-II,WT and OT-II,NCOR1 cKO^Cd4^ littermates. Histograms depict Vα2 expression on TCRβ^+^ CD4^+^ OT-II T cells. (**f**) Histogram depicts EGR2 expression in immature (TCRβ^hi^CD24^hi^) thymocytes of WT and NCOR1 cKO^Cd4^ mice. WT TCRβ^−/lo^ CD24^hi^ DP thymocytes were used as a negative staining control. (**g**) Histograms depict CD5 expression on DP (upper panel), CD4SP (middle panel) and TCRβ^hi^ CD8SP (lower panel) thymocytes in WT and NCOR1 cKO^Cd4^ mice. (**a**,**d**,**e**) Numbers indicate the percentage of cells in the respective quadrants. (**b**) Thick horizontal bars indicate the mean. (**b**,**c**) *P < 0.05, **P < 0.01 and ***P < 0.001 (unpaired two-tailed Student’s t-test). Data are representative (**a,d,e,f,g**) or show the summary (**b**,**c**) of 7–10 mice (**a**,**c**), 25–26 mice (**b**; except for CD4SP FOXP3^+^ cells, where n = 7 mice) and 5–6 mice (**d**,**e**,**f**,**g**) that were analyzed in 3–4 (**a**,**c**), 8 (**b**; except for CD4SP FOXP3^+^ cells: 3), 3 (**d**,**e**,**g**) and 2 (**f**) independent experiments.

**Figure 3:**
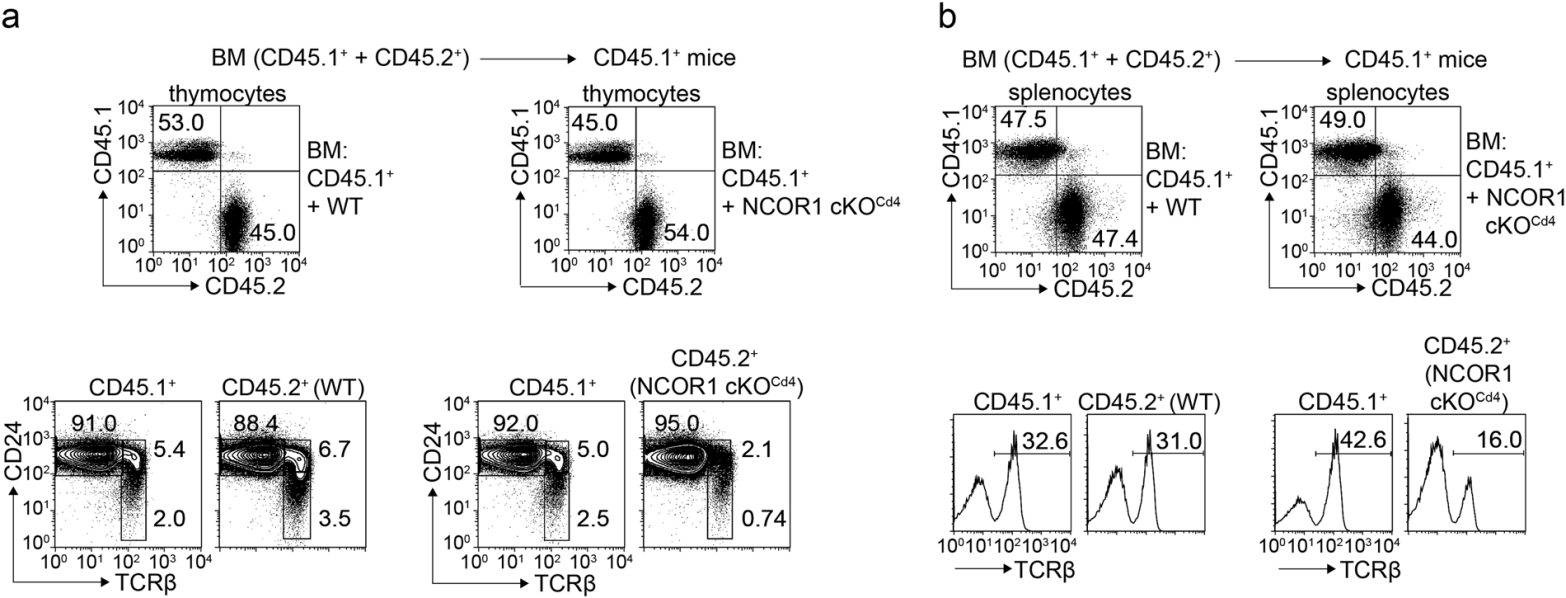
The reduction of SP cells in NCOR1 cKO^Cd4^ mice is T cell-intrinsic. Flow cytometry analysis of CD45.1 and CD45.2 expression (upper panels) on thymocytes (**a**) and splenocytes (**b**) isolated from BM chimeric CD45.1^+^ mice that received either *Ncor1* ^f/f^ (WT) or *Ncor1* ^f/f^ *Cd4*-Cre (NCOR1 cKO^Cd4^) CD45.2^+^ BM cells that had been mixed at a 1:1 ratio with wild-type (WT, CD45.1^+^) BM cells. The lower panels in (**a**) show CD24 and TCRβ expression on thymocytes pre-gated on CD45.1^+^ and CD45.2^+^ subsets, while the lower panels in (**b**) depicts TCRβ expression on splenocytes pre-gated on CD45.1^+^ and CD45.2^+^ subsets. Data are representative of 9–10 mice analyzed in 3 independent experiments.

To test whether developmental alterations caused the reduction in peripheral T cell numbers, thymocyte subsets in WT and NCOR1 cKO^Cd4^ mice were analyzed based on CD4, CD8, TCRβ and CD24 expression. This revealed that the percentages and cell numbers of CD4SP and TCRβ^hi^ CD8SP thymocytes were reduced in NCOR1 cKO^Cd4^ mice (Fig. 2a,b). There was a slight increase in the fraction of CD24^lo^ cells within the TCRβ^hi^ CD8SP population in the absence of NCOR1, while the distribution of CD24^hi^ to CD24^lo^ cells was normal within the CD4SP population (Fig. 2a,c). *Ncor1* was efficiently deleted from the DP stage on (Supplementary Figs S1b and S2b). NCOR1 protein was still detected in DP thymocytes, suggesting a slow turnover rate of NCOR1 protein in DP cells. However, NCOR1 protein almost completely disappeared in CD4SP cells (Supplementary Fig. S2b). Of note, in WT mice NCOR1 was expressed at higher levels in CD4SP than in DP thymocytes (Supplementary Fig. S2b), as previously observed^15^, suggesting an important role for NCOR1 during the DP to CD4SP transition. Like in the peripheral T cell population of NCOR1 cKO^Cd4^ mice, there was also a relative decrease of thymic FOXP3^+^ regulatory T cells within the already reduced CD4SP population (Fig. 2,b). The reduction in CD4SP and TCRβ^hi^ CD8SP thymocytes corresponded with a mild increase in the percentage of DP cells (Fig. 2a,b). However, the number of total thymocytes as well as of DP cells was similar between WT and NCOR1 cKO^Cd4^ mice (Fig. 2b). The percentages of mature CD4SP thymocytes and peripheral T cells were also reduced in NCOR1 cKO^Cd4^ mice transgenic for the MHC class II-restricted TCR OT-II (Fig. 2d,e). Of note, all TCR-transgenic OT-II,NCOR1 cKO^Cd4^ CD4^+^ T cells were TCR Vα2^+^ (Fig. 2e), indicating that CD4^+^ T cells were positively selected on the transgenic Vα2 chain. Furthermore, WT and NCOR1 cKO^Cd4^ TCRβ^hi^CD24^hi^ thymocytes upregulated the transcription factor EGR2 to a similar level (Fig. 2f) and TCRβ^hi^ SP cells that developed in NCOR1 cKO^Cd4^ mice showed a similar upregulation of CD5 as WT SP cells, suggesting no major alteration in TCR signaling strength during positive selection (Fig. 2g). Finally, the generation of either wild-type (CD45.1^+^) and *Ncor1* ^f/f^ (WT; CD45.2^+^) or wild-type (CD45.1^+^) and *Ncor1* ^f/f^ *Cd4-*Cre (NCOR1 cKO^Cd4^; CD45.2^+^) mixed bone marrow (BM) chimeric mice showed that the reduction in T cell numbers in the thymus and spleen was due to T cell-intrinsic effects and not due to secondary effects that affect mature T cell numbers (Fig. 3a,b).

### NCOR1 regulates the survival of positively selected TCRβ^hi^CD69^+/−^ thymocytes

**Figure 4:**
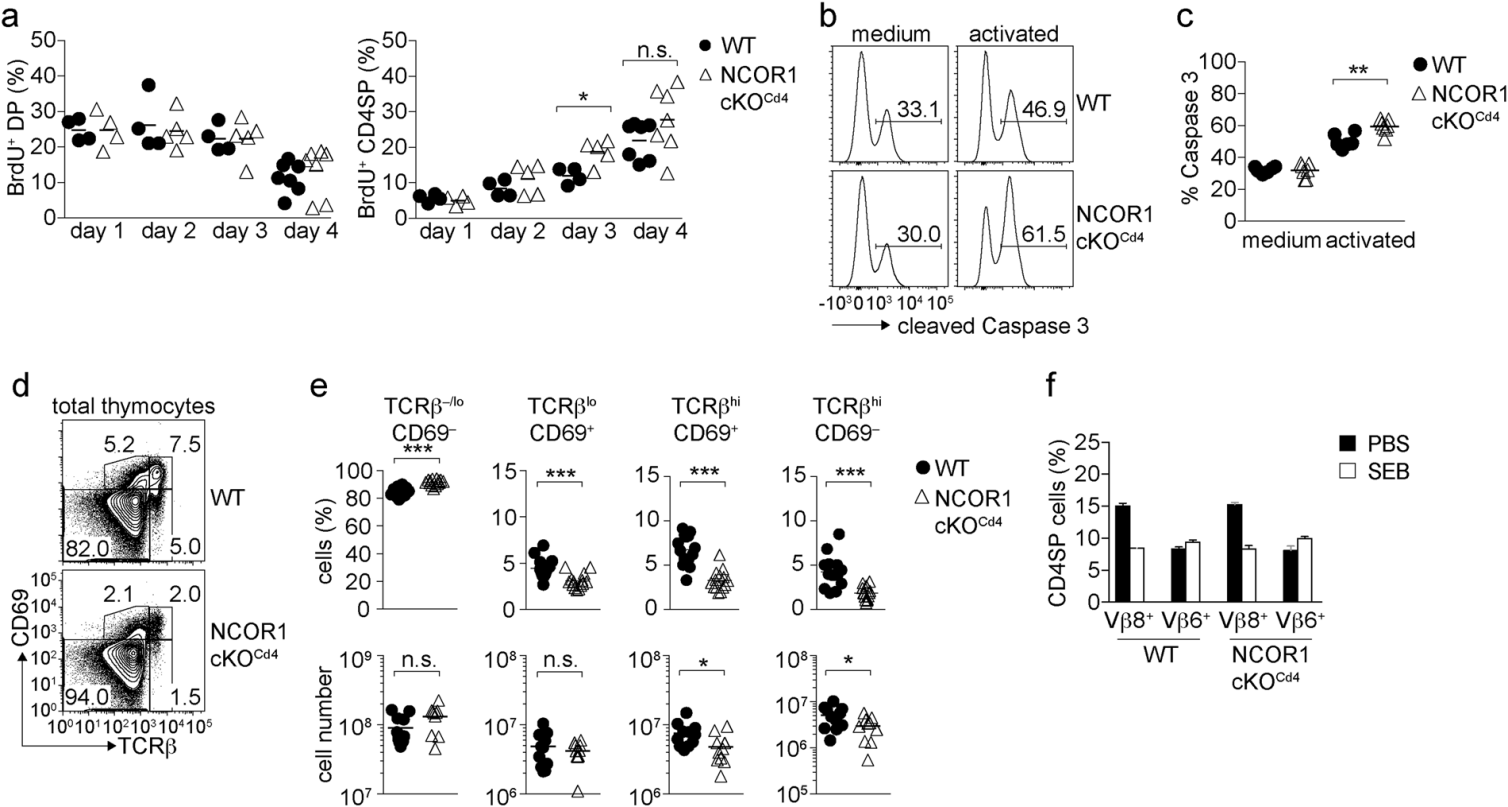
NCOR1 regulates the survival of positively selected thymocytes. (**a**) Summary shows the percentage of DP (left diagram) and CD4SP (right diagram) thymocyte subsets in WT and NCOR1 cKO^Cd4^ mice that have incorporated BrdU over the indicated time period. (**b**) Histograms depict cleaved caspase 3 in WT and NCOR1 cKO^Cd4^ thymocytes that were cultured for 24 h with medium only or activated with anti-CD3/anti-CD28. (**c**) The diagram shows the summary of experiments performed as described in (**b**). (**d**) Flow cytometry analysis of CD69 and TCRβ expression on thymocytes isolated from WT and NCOR1 cKO^Cd4^ mice. (**e**) Percentage and cell numbers of TCRβ^−/lo^CD69^−^, TCRβ^lo^CD69^+^, TCRβ^hi^CD69^+^ and TCRβ^hi^CD69^−^ thymocytes in WT and NCOR1 cKO^Cd4^ mice. (**f**) Diagram summarizing the percentages of Vβ8^+^ and Vβ6^+^ CD4SP subsets of WT and NCOR1 cKO^Cd4^ mice that have been injected with PBS or with SEB. (**b**,**d**) Numbers indicate the percentage of cells in the respective regions or quadrants. (**a**,**c**,**e**) Thick horizontal bars indicate the mean. *P < 0.05, **P < 0.01 and ***P < 0.001. Unpaired two-tailed Student’s t-test. Data are representative (**b**,**d**) or show the summary (**a**,**c**,**e**,**f**) of 4–6 mice (**a**), 6–7 mice (**b**,**c**), 14 mice (**d**,**e**; for percentages), 10–11 mice (**e**; for cell numbers), 5 (**f**; SEB injection) and 3 mice (**f**; PBS injection) that were analyzed in 2–3 (**a**), 2 (**b**,**c**,**f**) and 6–7 (**d**,**e**) independent experiments.

Next, we investigated in detail why SP thymocytes were reduced in the absence of NCOR1. *In vivo* BrdU labeling experiments showed that TCRβ^hi^CD24^lo^ CD4SP thymocytes developed with similar kinetics in NCOR1 cKO^Cd4^ mice in comparison to WT mice (Fig. 4a), indicating that there was no developmental block at the DP stage that results in a reduction of mature SP subsets. *Ex vivo*, NCOR1 cKO^Cd4^ thymocytes showed a higher fraction of cleaved caspase 3-positive cells after overnight culture in the presence of anti-CD3/anti-CD28, while in the absence of TCR stimulation the fraction of cleaved caspase 3-positive cells was similar between WT and NCOR1 cKO^Cd4^ (Fig. 4b,c). This suggests that the survival of thymocytes that received a TCR-mediated signal might be affected in the absence of NCOR1.

*In vivo*, positive and negative selection of thymocytes is dependent on persistent TCR signaling and associated with either the survival and maturation or the deletion of developing thymocytes, respectively. Thus, we investigated in more detail whether these processes are affected in NCOR1 cKO^Cd4^ mice. To analyze the differentiation of thymocytes during positive selection, we determined the dynamic expression pattern of TCRβ and CD69 that defines distinct stages of positive selection^23–25^: unsignaled (pre-selection) thymocytes (TCRβ^−/lo^CD69^−^); cells undergoing positive selection (TCRβ^lo^CD69^+^); postselection thymocytes (TCRβ^hi^CD69^+^) and mature SP thymocyte subsets (TCRβ^hi^CD69^−^) (Fig. 4d). In comparison to WT mice, there was a reduction in the percentages of signaled TCRβ^lo^CD69^+^ thymocyte subsets as well as strong reduction in the percentages and cell numbers of postselection TCRβ^hi^CD69^+^ and mature TCRβ^hi^CD69^−^ SP cells in the absence of NCOR1 (Fig. 4e). This indicated a significant loss of positively selected thymocytes in the absence of NCOR1 *in vivo*. To assess whether loss of NCOR1 affected also negative selection, we determined whether SEB superantigen-induced clonal deletion of SEB-reactive Vβ8^+^ CD4SP thymocytes is altered in NCOR1 cKO^Cd4^ mice^26^. There was a similar reduction of SEB-reactive Vβ8^+^ CD4SP cells in WT and NCOR1 cKO^Cd4^ mice, while SEB-non-reactive Vβ6^+^ CD4SP cells were not deleted upon injection of SEB (Fig. 4f). Together, these data indicated that NCOR1 cKO^Cd4^ SP cells are reduced as a consequence of an impaired survival of positively selected TCRβ^hi^CD69^+/−^ cells, rather than due to enhanced negative selection of NCOR1-deficient thymocytes.

### Mature NCOR1-null SP cells display elevated CD127 and BCL2 levels

**Figure 5:**
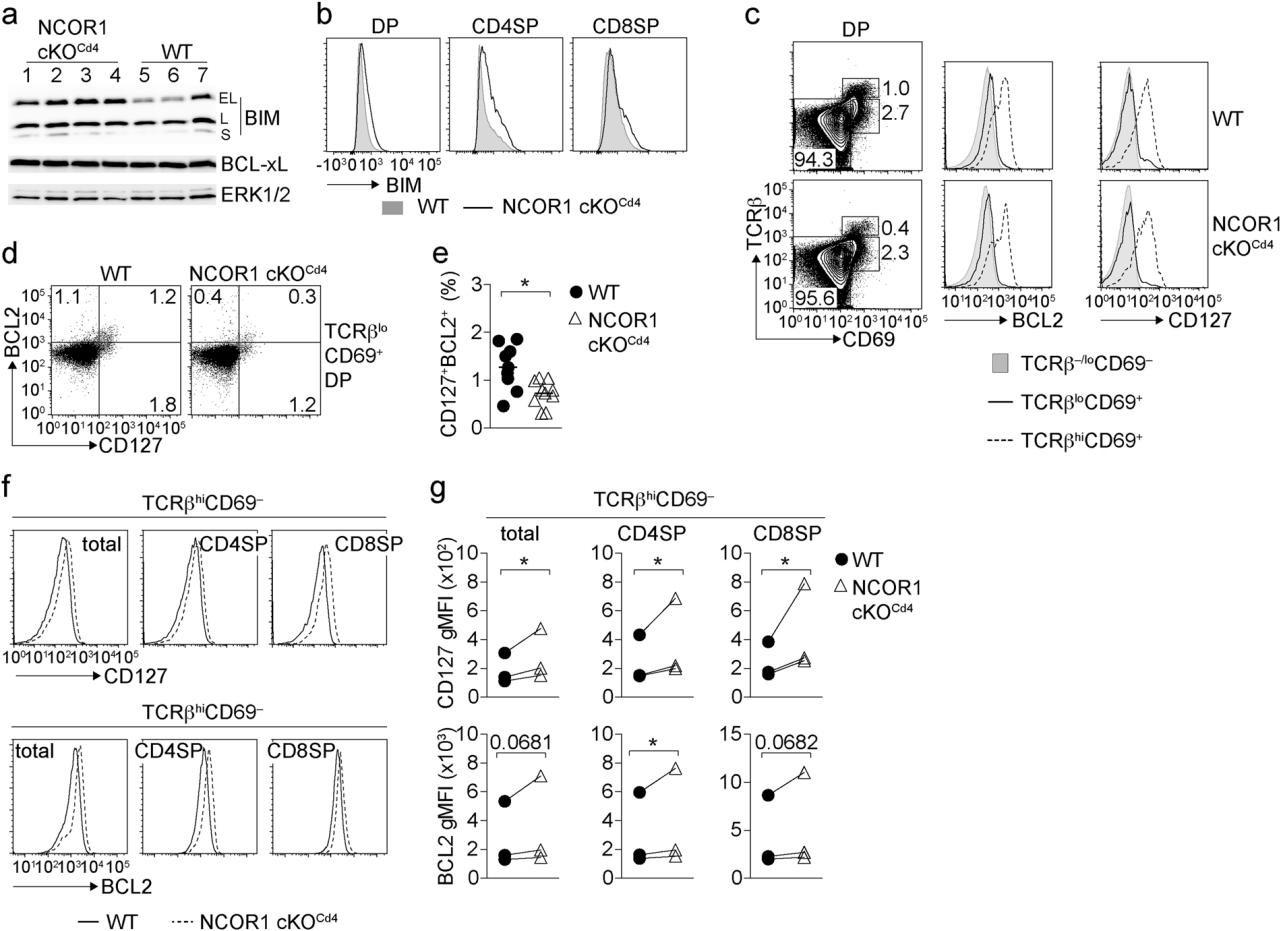
Mature NCOR1-null SP cells display elevated CD127 and BCL2 levels. (**a**) Immunoblot showing the three BIM isoforms (BIM_EL_, BIM_L_, BIM_S_) and BCL-xL expression in 4 independent NCOR1 cKO^Cd4^ (lane 1–4) and 3 independent WT (lane 5–7) thymocyte samples. Whole cell lysates from ~5 × 10^6^ cells were loaded and ERK1/2 was used as loading control. The pictures were cropped and uncropped pictures are shown in Supplementary Fig. 3. (**b**) Histograms depict pan BIM expression in WT and NCOR1 cKO^Cd4^ DP, CD4SP and CD8SP thymocytes. (**c**) Flow cytometry analysis of CD69 and TCRβ expression on DP thymocytes isolated from WT and NCOR1 cKO^Cd4^ mice. Histograms on the right show surface expression of CD127 and intracellular expression of BCL2 expression in the indicated subsets. (**d**) Flow cytometry analysis of BCL2 and CD127 expression on TCRβ^lo^CD69^+^ DP thymocytes isolated from WT and NCOR1 cKO^Cd4^ mice. (**e**) The diagram indicates the percentage of BCL2^+^CD127^+^ TCRβ^lo^CD69^+^ DP thymocytes. (**f**) Histograms depict CD127 (upper panel) and BCL2 (lower panel) expression in TCRβ^hi^CD69^−^ thymocyte subsets (total, CD4SP and CD8SP) in WT and NCOR1 cKO^Cd4^ mice. (**g**) The diagrams indicate geometric mean fluorescence intensity (gMFI) of CD127 and BCL2 expression on the particular subset, and show the summary of 3 independent experiments (2–5 mice were analyzed per group in an experiment, average gMFI for each experiment is shown). The lines connect samples analyzed in the same experiment. (**e**) *P < 0.01, unpaired two-tailed Student’s t-test. (**g**) *P < 0.01, n.s., not significant, one sample t-test (columns statistics; WT was set as 1 and relative NCOR1 cKO^Cd4^ values were calculated, diagrams show absolute gMFI levels for each experiment). Data are representative (**a**,**b**,**c**,**d**,**f**) or show the summary (**e**,**g**) of 7 (**a**), 7–8 (**b**), 9–10 (**d**,**e**) mice that were analyzed in 2 (**a**,**b**) and 3 (**c**,**d**,**e**) independent experiments. Histograms (**c**,**f**) are representative of 9–10 mice analyzed in 3 independent experiments.

The survival of DP and SP thymocytes is dependent on the balanced expression of the pro-apoptotic protein BIM and the pro-survival factors BCL-xL and BCL2 that are dynamically expressed during thymocyte development^27–29^. Immunoblot analysis of total NCOR1 cKO^Cd4^ thymocytes revealed higher expression levels of the pro-apoptotic factor BIM, in particular the BIM_EL_ isoform (Fig. 5a), which was due to increased BIM expression both in DP as well as CD4SP thymocytes and, to a lower degree, in CD8SP subsets (Fig. 5b). In contrast, the expression of BCL-xL, which is important for the survival of DP thymocytes but not of SP cells^27,30^, was not changed (Fig. 5a), indicating that there is no increase in BCL-xL expression to compensate for higher BIM levels. Positive selection correlates with an upregulation of the IL-7 receptor (IL-7R), a cytokine receptor which triggers the induction of BCL2 to ensure the survival of positively selected cells^31^. A detailed analysis of DP thymocytes revealed that TCRβ^hi^CD69^+^ DP cells present in NCOR1 cKO^Cd4^ mice upregulated BCL2 and CD127 similar to WT TCRβ^hi^CD69^+^ DP thymocytes (Fig. 5c and Supplementary Fig. S4). However, NCOR1 cKO^Cd4^ TCRβ^lo^CD69^+^ DP cells showed a 2-fold reduction in the fraction of cells that expressed both the IL-7Rα chain (CD127) and BCL2 (Fig. 5d,e). This suggests a reduced survival of TCR-triggered TCRβ^lo^CD69^+^ DP thymocyte subsets in NCOR1 cKO^Cd4^ mice. A close examination of CD127 and BCL2 expression at later stages of thymocyte development revealed that positively selected mature TCRβ^hi^CD69^−^ total SP, CD4SP and CD8SP cells displayed higher CD127 expression levels (increase gMFI for total SP: 45 ± 11%; CD4SP: 46 ± 12%; CD8SP: 74 ± 27%) and mildly increased levels of BCL2 (increase gMFI for total SP: 22 ± 12%; CD4SP: 20 ± 9%; CD8SP: 18 ± 9%) in the absence of NCOR1 (Fig. 5f,g). These data indicate that mature NCOR1 cKO^Cd4^ SP cells that survived displayed elevated CD127 and BCL2 levels, which might partially compensate for the increased BIM expression levels in NCOR1 cKO^Cd4^ SP thymocytes.

### Transgenic expression of BCL2 rescues the generation of NCOR1-null SP thymocytes

**Figure 6:**
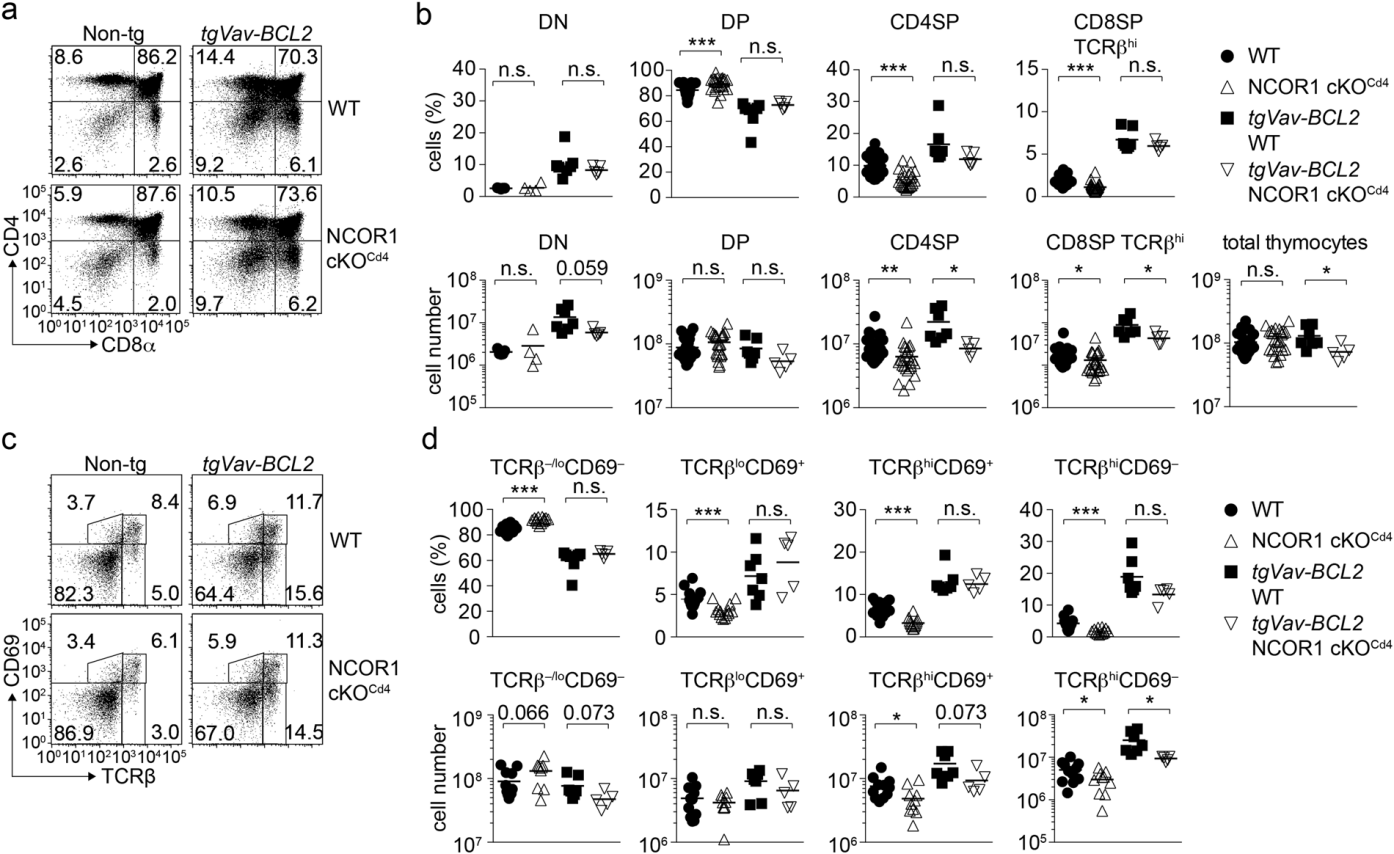
Transgenic expression of BCL2 rescues the generation of NCOR1 cKO^Cd4^ SP thymocytes. (**a**) Flow cytometry analysis of CD4 and CD8 expression on thymocytes isolated from WT and NCOR1 cKO^Cd4^ mice that are non-transgenic (left panel) or transgenic for *Vav-BCL2* (right panel). (**b**) Percentages (upper panel) and cell numbers (lower panel) of DN, DP, CD4SP and TCRβ^hi^ CD8SP thymocytes in WT and NCOR1 cKO^Cd4^ mice that are non-transgenic or transgenic for *Vav*-*BCL2* are shown. (**c**) Flow cytometry analysis of CD69 and TCRβ expression on thymocytes isolated from WT and NCOR1 cKO^Cd4^ mice that are non-transgenic (left panel) or transgenic for *Vav-BCL2* (right panel). (**d**) Percentages (upper panel) and cell numbers (lower panel) of TCRβ^−/lo^CD69^−^, TCRβ^lo^CD69^+^, TCRβ^hi^CD69^+^ and TCRβ^hi^CD69^−^ thymocytes in WT and NCOR1 cKO^Cd4^ mice that are non-transgenic and transgenic for *Vav*-*BCL2* are indicated. (**a**,**c**) Numbers indicate the percentage of cells in the respective quadrants or regions. (**b**,**d**) Thick horizontal bars indicate the mean; *P < 0.05, **P < 0.01 and ***P < 0.001, n.s., not significant; unpaired two-tailed Student’s t-test. Data are representative (**a**,**c**) or show the summary (**b**,**d**) of 9–15 (for non transgenic mice; non-tg) and 5–9 (for tg*Vav-BCL2* mice) mice that were analyzed in 4–7 independent experiments. (**b**,**d**) The data for WT and NCOR1 cKO^Cd4^ mice in (**b**; except DN subsets) and (**d**) were already shown in Figures 2b and 4e, respectively.

The anti-apoptotic factor BCL2 is important for the survival of positively selected cells^31^. Mature TCRβ^hi^CD69^−^ SP thymocytes that survived positive selection in the absence of NCOR1 expressed mildly increased levels of BCL2 (Fig. 5f,g). Therefore, we next investigated whether the loss of CD69^+^ NCOR1-null thymocytes can be rescued by enforced expression of BCL2. As previously reported^32^, *Vav* promotor-driven transgenic expression of (human) BCL2 increases the percentages and numbers of DN, CD4SP and CD8SP thymocytes in WT mice with a corresponding decrease in DP thymocytes (Fig. 6a,b). As a consequence, there is also an increase in TCRβ^lo^CD69^+^, TCRβ^hi^CD69^+^ and mature SP TCRβ^hi^CD69^−^ subsets (Fig. 6c,d). Similar changes in DN, DP, CD4SP and TCRβ^hi^ CD8SP thymocytes subsets due to transgenic BCL2 expression were also observed on a NCOR1 cKO^Cd4^ background (Fig. 6a,b). Further, upon transgenic BCL2 expression in NCOR1 cKO^Cd4^ mice, the percentages of TCRβ^−/lo^CD69^−^, TCRβ^lo^CD69^+^, TCRβ^hi^CD69^+^ and mature SP TCRβ^hi^CD69^−^ subsets were similar to WT mice (Fig. 6c,d), suggesting that transgenic BCL2 overexpression restored the percentages of TCRβ^hi^ cells within the NCOR1 cKO^Cd4^ thymocyte population. The ability of BCL2 to rescue NCOR1 cKO^Cd4^ thymocytes strongly supports our findings that signaled CD69^+^ thymocytes are lost due to apoptosis rather than due to a developmental block at the onset of positive selection or due to increased negative selection. In contrast to transgenic BCL2, *Lck* promotor-driven transgenic expression of BCL-xL^33^ in NCOR1 cKO^Cd4^ mice did not rescue the percentages of TCRβ^hi^CD69^+^ and TCRβ^hi^CD69^−^ thymocytes to levels observed in tg*BCL-xL*,WT mice (Supplementary Fig. S5). Since BCL-xL is a pro-survival factor important for the lifespan of DP thymocytes but dispensable for thymocyte survival during positive selection and maturation^27,30^, these data suggest that loss of NCOR1 affects the survival of positively selected thymocytes.

## Discussion

In this study we provide genetic evidence that NCOR1 is essential for the generation of the peripheral T cell pool by regulating the survival of positively selected TCRβ^hi^CD69^+/−^ thymocytes. In our study we also observed that DP cells were present at similar numbers in WT and NCOR1 cKO^Cd4^ mice. Of note, NCOR1 protein levels in NCOR1 cKO^Cd4^ DP thymocytes were similar to those in WT DP cells despite an efficient genomic deletion of *Ncor1*. This indicates a slow turnover of NCOR1 protein and precluded conclusions about the role of NCOR1 in DP thymocytes. However, our data indicate an important role for NCOR1 beyond the DP stage, since there was a gradual decline of the percentages of NCOR1-null thymocytes from the CD69^+^ stage on during the DP to SP transition, which led to a significant reduction in cell numbers of TCRβ^hi^CD69^+/−^ cells. The DP to SP transition is also accompanied by an upregulation of NCOR1, since WT CD4SP cells expressed higher NCOR1 levels in comparison to WT DP cells. It is not known at which TCRβ^lo/hi^CD69^+/−^ stage NCOR1 is upregulated. This process might occur gradually, potentially leading to a progressive increase in phenotypic alterations from the onset of positive selection to the mature SP stage in the absence of NCOR1. BrdU labeling studies showed a similar appearance of BrdU^+^ cells within the SP thymocyte population in NCOR1 cKO^Cd4^ mice. In addition, MHC class II-restricted OT-II TCR transgenic NCOR1 cKO^Cd4^ CD4^+^ T cells, which were also reduced in the absence of NCOR1, displayed a similar expression of the transgenic Vα2 chain. Thus, it is unlikely that a block in positive selection or changes in the TCR signaling strength caused the reduction of SP cells in the absence of NCOR1. This is also supported by the observation that CD5 expression, which parallels the avidity or signaling intensity of the positively selecting TCR–MHC-ligand interaction^34^, is similar in WT and NCOR1 cKO^Cd4^ SP cells. Our data rather indicate that NCOR1 is essential for the efficient survival of positively selected TCRβ^hi^CD69^+/−^ thymocytes. They further suggest that the first phenotypic alterations in NCOR1 cKO^Cd4^ might be initiated already in TCRβ^lo^CD69^+^ cells undergoing positive selection, consistent with the observation that NCOR1 cKO^Cd4^ signaled TCRβ^lo^CD69^+^ DP thymocytes showed a lower fraction of cells that upregulated both CD127 (IL-7Rα chain) and BCL2 in comparison to WT thymocytes. Together with the elevated expression of BIM, it is likely that a change in the relative abundance of BIM and BCL2 leads to reduced survival of positively selected SP thymocytes^35,36^. We also observed that mature NCOR1-null TCRβ^hi^CD69^−^ SP thymocytes that survived displayed higher expression levels of CD127 and BCL2 in comparison to mature WT SP cells, suggesting that high levels of CD127 as well as elevated BCL2 expression are sufficient to balance enhanced BIM expression. In line with this data is our observation that transgenic expression of BCL2 restored the percentages of TCRβ^lo/hi^CD69^+^ thymocytes to levels observed in WT mice and as a consequence also the percentage of SP cells. Cell numbers of CD4SP and CD8SP in tg*Vav*-*BCL2*,NCOR1 cKO^Cd4^ mice were also increased but still significantly lower in comparison to tg*Vav*-*BCL2*,WT control mice, suggesting that the overexpression of BCL2 does not fully complement the survival defect of NCOR1-null SP thymocytes.

Of note, we observed high BIM expression levels in NCOR1 cKO^Cd4^ mice despite residual NCOR1 expression, suggesting that subtle changes in NCOR1 levels might be sufficient to induce BIM expression. However, this did not affect the survival of pre-selection CD69^−^ DP thymocytes, which were present at similar numbers in WT and NCOR1 cKO^Cd4^ mice. This finding suggests that either the increase in BIM is not sufficient to overcome the pro-survival capacity of BCL-xL in DP thymocytes, or that BIM is not activated by posttranslational modifications, such as JNK-dependent Thr112 phosphorylation^37^. Moreover, transgenic expression of BCL-xL, which is important for the survival of DP thymocytes^27,30^, in NCOR1 cKO^Cd4^ mice did not lead to an increase in the percentages of TCRβ^hi^CD69^+^ cells as well as of TCRβ^hi^CD69^−^ SP cells to values observed upon expression in WT mice, showing that BCL-xL did not rescue the phenotype. WT CD4 SP cells expressed higher NCOR1 levels in comparison to WT DP cells, thus TCR triggering of DP cells might induce the upregulation of NCOR1 expression and protection from apoptosis during the DP to SP transition. This suggests that signals induced by TCR triggering of DP cells make them susceptible to BIM-mediated apoptosis in the absence of NCOR1 upregulation. In addition, *ex vivo* anti-CD3-stimulated NCOR1-null thymocytes displayed increased levels of cleaved caspase 3 in comparison to WT cells, while there was no difference without TCR triggering. However, SEB-induced negative selection was not changed in the absence of NCOR1, pointing towards a role for NCOR1 rather in the regulation of cell survival during positive selection and SP development but not in lowering the apoptotic threshold during negative selection. The mechanism by which loss of NCOR1 leads to the upregulation of BIM is not known and whether NCOR1 directly regulates *Bcl2l11* gene (encoding for BIM) expression remains to be determined. The repressive activity of NCOR1-containing complexes is mediated via the recruitment of histone deacetylases (HDACs)^2,6^. Interestingly, HDAC inhibitors developed for cancer therapy do exert their activity in part via increasing the expression of BIM^38,39^ suggesting a potential molecular mechanism of how NCOR1 might repress BIM transcription. However, since NCOR1 is also linked with metabolic homeostasis in other cell lineages such as adipocytes^9^, muscle cells^8^ and macrophages^10^, we cannot exclude at present that the upregulation of BIM is a consequence of alterations beyond a direct transcriptional regulation by NCOR1.

Among all the HDAC family members, NCOR1 mainly associates with HDAC3 to repress target gene transcription^2,6^. Interestingly, the observed phenotype in the absence of NCOR1 is reminiscent to some of the phenotypes observed in mice with an early deletion of HDAC3 mediated by *Cd2*-iCre (HDAC3 cKO^Cd2^)^21^ or *Lck*-Cre (HDAC3 cKO^Lck^)^20^. It is tempting to speculate that NCOR1 and HDAC3 might partially act together in regulating SP thymocyte survival. In comparison to NCOR1 cKO^Cd4^ mice, the reduction in SP cells is much more severe in the absence of HDAC3, which might be the consequence of the early deletion of HDAC3 during thymocyte development in those studies. Since there is, as discussed above, residual NCOR1 protein expression in NCOR1 cKO^Cd4^ DP thymocytes, it is also possible that this might prevent a more severe drop in SP thymocyte numbers. However, qualitative differences in the phenotype of HDAC3-null and NCOR1-null thymocytes suggest also unique functions for each molecule in the regulation of SP thymocyte development. For HDAC3 cKO^Cd2^ mice, it was shown that signaled HDAC3-null DP thymocytes failed to downregulate RORγt expression during positive selection. The prolonged expression of RORγt, which correlated with increased acetylation of the *Rorc* gene locus (encoding RORγt), has been linked to the observed block in positive selection in the absence of HDAC3^21^. On the contrary, NCOR1 cKO^Cd4^ thymocytes downregulated RORγt in a similar manner as positively selected WT thymocytes (Supplementary Figs S6a and S6b). Further, HDAC3 cKO^Cd2^ semimature TCRβ^hi^CD24^+^ CD4SP thymocytes do not properly upregulate CD127 and EGR2^21^, which both have been shown to induce BCL2 expression^31,40^. This is in contrast to semimature TCRβ^hi^CD24^+^ thymocytes that developed in NCOR cKO^Cd4^ mice, which expressed similar levels of EGR2. In addition, BIM expression was normal in HDAC3 cKO^Cd2^ TCRβ^hi^CD24^+^ CD4SP thymocytes^21^, while BIM was upregulated in SP thymocytes in the absence of NCOR1. Differences in the function of NCOR1 and HDAC3 are also underscored by the comparison of the phenotypes of NCOR1 cKO^Cd4^ and HDAC3 cKO^Cd4^ mice. In contrast to NCOR1, late deletion of HDAC3 in DP thymocytes using *Cd4*-Cre (HDAC3 cKO^Cd4^) does not lead to major changes in the generation of conventional CD4SP and CD8SP cells^18,19^. However, peripheral CD4^+^ and CD8^+^ T cell numbers are almost 10-fold and 6-fold decreased in HDAC3 cKO^Cd4^ mice, respectively^18,19^. By using *Cd4*-Cre-mediated deletion, it has also been shown that HDAC3 is important for post-thymic T cell maturation. The majority of peripheral HDAC3 cKO^Cd4^ T cells are recent thymic emigrants blocked in their functional maturation, and are subsequently eliminated by the complement system due to a defect in sialic acid modification and binding of IgM and complement proteins^19^. Further, in comparison to WT cells peripheral HDAC3-null T cells express lower levels of the complement inhibitor CD55^19^, a marker found on matured naïve T cells^41^. In NCOR1 cKO^Cd4^ mice, such a severe reduction in peripheral T cell numbers was not observed (T cells were only 2-fold reduced in the absence of NCOR1) and expression of CD55 was similar on naive WT and NCOR1 cKO^Cd4^ CD4^+^ T cells (Fig. S6c). Together, these data indicate that NCOR1 and HDAC3 might not synergistically regulate the survival of SP thymocytes. The differences in how the loss of NCOR1 and HDAC3 affects the dynamic expression of EGR2, CD127, BCL2 and BIM during positive selection and in positively selected thymocytes, as well as the important role for HDAC3 in T cell maturation  clearly highlight unique functions for NCOR1 and HDAC3 in the regulation of SP thymocyte survival and the generation of the peripheral T cell compartment. NCOR1 and HDAC3 might be integrated in the same as well as in different transcription factor complexes at a given developmental stage and thus regulate common but also unique target genes at crucial developmental checkpoints. NCOR1 interacts with nuclear hormone receptors and with several BTB-ZF transcription factors^3,5^, and NCOR1 associates with other HDACs such as HDAC1, 4, 5 and 7^6^. Thus, NCOR1 might recruit different repressor complexes via different types of transcription factors to target genes independently of HDAC3, which might have an impact on the survival of positively selected thymocytes. Moreover, NCOR1 has been linked to the metabolic regulation of cells^3^ and thus changes in these processes might also lead to reduced numbers of SP thymocytes. Further studies including RNA-seq and ChIP-seq experiments with WT, HDAC3-null and NCOR1-null thymocytes are required to dissect in more detail NCOR1 and HDAC3 mediated transcriptional networks that control T cell development.

In summary, our study identified NCOR1 as an important factor controlling T cell homeostasis by regulating the survival of positively selected thymocytes and thus the size of the peripheral T cell pool.

## Methods

### Animal models

Animal experiments were evaluated by the ethics committee of the Medical University of Vienna and approved by the Federal Ministry for Science and Research, Vienna, Austria (GZ:BMWF-66.009/0057-II/10b/2010; GZ:BMWF-66.009/58-II/10b/2010; GZ:BMWF-66.009/0103-WF/II/3b/2014; GZ:BMWF-66.009/0105-WF/II/3b/2014). Animal husbandry and experimentation was performed under the national laws (Federal Ministry for Economy and Science, Vienna, Austria) according to the guidelines of the Federation of Laboratory Animal Science Associations (FELASA), which match that of Animal Research: Reporting *In Vivo* Experiments (ARRIVE). Mice carrying a loxP-flanked (floxed) *Ncor1* allele have been described^8^. *Cd4-*Cre mice were kindly provided by Dr. Chris Wilson. OT-II TCR transgenic mice were kindly provided by Dr. Stoiber-Sakaguchi (Medical University of Vienna). BCL2 transgenic mice^32^ were kindly made available by Dr. Jerry M. Adams and provided by Dr. Veronika Sexl (University of Veterinary Medicine, Vienna). BCL-xL transgenic mice^33^ were kindly provided by the late Stanley J. Korsmeyer. All mice analyzed were 6–8 weeks of age and of mixed sex unless otherwise stated. Littermate controls were used for the flow cytometry analysis within one experiment.

### Generation of mixed bone marrow chimeric mice

Mixed BM chimeric mice were generated as previously described^42^. Six to eight weeks after transplantation, the reconstituted mice were sacrificed and analyzed by flow cytometry (LSRII or LSRFortessa, BD Biosciences).

### Flow cytometry analysis

Thymii and spleens of mice were removed and placed into 6 well tissue culture plates containing staining buffer (2% v/v FCS in PBS). Single cell suspensions were made by passage of the tissue through a 70μm nylon cell strainer (Corning). Erythrocytes were removed with Pharmlyse (BD Biosciences). Cell suspensions were washed once with staining buffer and 2–5 × 10^6^ cells were incubated with Fc-block (BD Pharmingen) and stained for 30 min with fluorophore-conjugated antibodies against various surface molecules. After the staining reaction, cells were washed once with staining buffer and acquired through LSRII or LSRFortessa (BD Biosciences) flow cytometer or prepared for intracellular staining. Data were analyzed using FlowJo software (Tree Star). Doublets were excluded from analysis.

### Cell isolation for *Ncor1* deletion PCR

CD4^+^ and CD8^+^ T cells were isolated from WT and NCOR1 cKO^Cd4^ spleens by negative depletion of B cells, NK cells and myeloid cells using biotinylated antibodies (anti-mouse B220, NK1.1, CD11c, CD11b, Gr-1; eBioscience or BD Biosciences) prior to sorting. Naive T cells were purified as CD4^+^ or CD8^+^ CD62L^+^CD44^−^CD25^−^ cells. DN, DP, CD4SP and CD8SP TCRβ^hi^ thymocyte subsets were sorted from WT and NCOR1 cKO^Cd4^ thymii by using anti-CD4, anti-CD8α and anti-TCRβ antibodies. Subsequently, 1 × 10^5^ cells were lysed in tail lysis buffer for 2 hrs at 55 °C. The *Ncor1* deletion PCR was performed from the lysate.

### Intracellular transcription factor staining

Intracellular detection of FOXP3, EGR2 and RORγt was performed with the FOXP3 Transcription Factor staining buffer set (eBioscience) according to the manufacturer’s instructions. Intracellular BCL2 staining was performed sequentially with Cytofix Fixation Buffer and Perm/Wash Buffer (both from BD Biosciences) according to the manufacturer’s instructions. Viability dye (eBioscience) was used to exclude dead cells from the analysis.

### Intracellular BIM staining

Total thymocytes (8 × 10^6^) were fixed with Cytofix Fixation buffer (BD Biosciences) and permeabilized with Perm/Wash buffer (BD Biosciences). Cells were incubated with rat anti-BIM in Perm/Wash buffer. Subsequently, cells were washed once and incubated with R-Phycoerythrin (R-PE) donkey anti-rat IgG in permeabilization buffer to reveal intracellular BIM staining by flow cytometry, T cell surface marker staining was performed following intracellular BIM staining. Viability dye (eBiosciences) was used to exclude dead cells from the analysis.

### Antibodies used for flow cytometry

The following antibodies were used: from eBioscience: Anti-BrdU (clone Bu20A), anti-CD127 (A7R34), anti-CD4 (RM4–5), anti-CD8α (53–6.7), anti-EGR2 (erongr2), anti-FOXP3 (FKJ-16s), anti-TCR Vα2 (B20.1), anti-TCRβ (H57-597), anti-Vβ6 TCR (RR4-7), anti-Vβ8.1/Vβ8.2 TCR (Kj16-1333); from BD Biosciences: anti-active caspase 3 (C92-105), anti-CD4 (Gk1.5), anti-CD24 (M1/69), anti-CD16/CD32 (2.4G2), anti-CD44 (IM7), anti-CD45.1 (A20), anti-CD45.2 (104), anti-CD45R/B220 (RA3-6B2), anti-CD5 (53-7,3), anti-CD62L (MEL-14), anti-CD69 (H1.2F3), anti-RORγt (Q31-378); from Biolegend: anti-BCL2 (BCL/10C4).

### *In vivo* SEB injections

Mice were injected (i.p.) with 10 μg staphylococcal enterotoxin B (SEB) (Sigma-Aldrich) (0.1 mg/ml in sterile PBS) on days 0, 2 and 4. Mice were sacrificed on day 7 and thymocytes were isolated, stained for surface markers and analyzed by flow cytometry.

### Cleaved caspase 3 staining

Total thymocytes (3 × 10^6^ cells/well of a 24 well plate) were activated for 24 h with plate-bound anti-CD3ε (1 μg/ml) and anti-CD28 (3 μg/ml) in 1 ml T cell medium (RPMI, GlutaMAX-I supplemented with 10% v/v FCS, antibiotics and 2-mercapthoethanol; all from Sigma-Aldrich) or left non-activated. After harvesting, thymocytes were stained with the various antibodies. Viability dye (eBioscience) was added before the surface staining to detect dead cells during the analysis. Intracellular cleaved caspase 3 staining was performed with Cytofix Fixation Buffer and Perm/Wash Buffer (BD Biosciences) according to the manufacturer’s instructions.

### BrdU incorporation assay

Mice were injected (i.p.) with 1 mg BrdU (eBioscience or BD Pharmingen) (10 mg/ml in PBS). Thymocytes were isolated 24, 48, 72 or 96 hrs later and stained for surface markers. Subsequently, BrdU was detected with eBioscience BrdU Staining Kit for Flow cytometry or BD Pharmingen APC BrdU Flow Kit according to the manufacturer’s protocol.

### Immunoblot analysis to detect BIM, BCL-xL and ERK1/2 expression

Thymocytes (5 × 10^6^) were lysed in 25 μl RIPA buffer (25 mM Tris pH 8.0, 150 mM NaCl, 1.0% Triton-X, 0.1% SDS, 1% sodium deoxycholate, 1 mM EDTA) supplemented with complete protease inhibitors (Roche) and phosphatase inhibitors 1 mM Na_3_VO_4_ and 1 mM NaF. Proteins were separated on 12% SDS-polyacrylamide gels and electroblotted on AmershamTM HybondTM-ECL nitrocellulose membranes (GE Healthcare) according to standard protocols. Membranes were blocked in 5% (w/v) milk in PBST for 1 h and incubated with primary antibodies overnight. The following primary antibodies were used: rat anti-BIM (3C5/WEHI/Alexis), rabbit anti-BCL-xL (clone 54H6, Cell Signalling) and rabbit anti-ERK1/2 (clone 9102, Cell Signaling). All primary antibodies were diluted in 5% (w/v) milk in PBST. HRP-conjugated rabbit anti-rat IgG and goat anti-rabbit IgG (JacksonImmunoResearch Laboratories) were used as secondary antibodies. Immunoblots were developed using Western Bright ECL Spray (Advansta) and HRP chemiluminescence signals were detected with a Fujifilm LAS-4000 image analyzer (GE Healthcare) and analyzed with the Multi Gauge V3.0 software.

### Immunoblot analysis to detect NCOR1 and α-Tubulin expression

Total thymocytes as well as sorted DP and CD4SP thymocytes (2 × 10^6^) were lysed in 25 μl Carin Lysis buffer (20 mM Tris-HCl pH 8.0, 138 mM, NaCl, 10 mM EDTA, 1% Nonidet P-40, 10% glycerol) supplemented with complete protease inhibitors (Roche) and phosphatase inhibitors Na_3_VO_4_ (1 mM) and NaF (1 mM). Proteins were separated on 6% SDS-polyacrylamide gels and electroblotted on AmershamTM HybondTM-ECL nitrocellulose membranes (GE Healthcare) according to standard protocols. Membranes were blocked in 3% (w/v) milk in PBST for 1 h and incubated with primary antibodies for 3 hrs on room temperature. The following primary antibodies were used: rabbit polyclonal anti-NCOR1 (PA1-844A, Invitrogen), goat anti-NCOR1 (clone C-20, Santa Cruz) and mouse anti-α-Tubulin (clone DM1A, Sigma Aldrich). All primary antibodies were diluted in 3% (w/v) milk in PBST. HRP-conjugated goat anti-rabbit IgG and mouse anti-goat IgG (JacksonImmunoResearch Laboratories) were used as secondary antibodies. Immunoblots were developed using either Western Bright ECL Spray (Advansta) or Clarity Max Western ECL substrate (Bio Rad). HRP chemiluminescence signals were detected with a Fujifilm LAS-4000 image analyzer (GE Healthcare) and analyzed with the Multi Gauge V3.0 software.

### Primers

The following primers were used: *Ncor1 floxed and Δ allele:* Ncor1 (#31), 5′- TTG GCC TTG GAG TAA ATG CTG TGA G; Ncor1 (#32), 5′- GGA AAC TAC CTA CCT GAA TCC ATG G; Ncor1 (#29) 5′- GAA CTA AGG ACA GGA AGG TAC AGG G. *tgLck-BCL-xL*: BCL-xL TG fwd, 5′- GCA TTC AGT GAC CTG ACA TC; BCL-xL TG rev, 5′-CTG AAG AGT GAG CCC AGC AGA CC. *TgVav-BCL2:* Vav BCL2 #1, 5′- ACG GTG GTG GAG GAG CTC TTC; Vav BCL2 #2, 5′- AAA ACC TTC CCA CAC CTC CCC CTG AA. *Cd4*-Cre: CD4-Cre F2, 5′-ACG GTA GTT TGT CTT TGG CAC C, CD4-Cre R2, 5′- CTT CTT GGG TGC CAT GCT CG.

### Statistical analysis

No statistical methods were used to predetermine the sample size. The data shown indicate the mean. All experiments that required a statistical analysis were performed at least three times. The statistical analyses were performed using Prism Software (GraphPad Inc). As indicated in each figure legend, P-values were calculated with either an unpaired two-tailed Student’s t test (a normal distribution of data points was assumed; variances were assessed and if necessary an unpaired t-test with Welch’s correction was applied) or with an one sample t-test (columns statistics; Fig. 5g and Supplementary Fig. S4). No data were excluded and no specific randomization of animals or blinding of investigators was applied.

## Electronic supplementary material

Supplementary Information
