# Supplementary material for: The corepressor NCOR1 regulates the survival of single-positive thymocytes
Source: Sci Rep. 2017 Nov 21;7:15928. doi: 10.1038/s41598-017-15918-0 (PMC5698297; doi:10.1038/s41598-017-15918-0)
Supplement: Supplementary file 1 — Supplementary Information [file 41598_2017_15918_MOESM1_ESM.pdf]

Supplementary Information for the manuscript:
The corepressor NCOR1 regulates the survival of single-positive thymocytes

Running title: NCOR1 regulates the survival of SP thymocytes

Authors: Lena Müller[1], Daniela Hainberger[1], Valentina Stolz[1], Patricia Hamminger[1], Hammad Hassan[1,4], Teresa Preglej[1], Nicole Boucheron[1], Shinya Sakaguchi[1], G. Jan Wiegers[2], Andreas Villunger[2], Johan Auwerx[3], Wilfried Ellmeier[1]

[1]Division of Immunobiology, Institute of Immunology, Center for Pathophysiology, Infectiology and Immunology, Medical University of Vienna, 1090 Vienna, Austria.
[2]Innsbruck Medical University, Biocenter, Division of Developmental Immunology, Innsbruck, Austria.
[3]Ecole Polytechnique Fédérale de Lausanne, Laboratory of Integrative and Systems Physiology, Lausanne, Switzerland
[4]Current address:
Dept. of Biochemistry (Shankar Campus), Abdul Wali Khan University (AWKUM) Mardan, KPK, Pakistan.

Correspondence should be addressed to W.E. (wilfried.ellmeier@meduniwien.ac.at).

Supplementary Figure 1 shows the deletion efficiency of *Ncor1* in peripheral T cells and thymocyte subsets using PCR and NCOR1 protein expression using immunoblotting.

Supplementary Figure 2 shows pictures of the uncropped agarose gels and immunoblots shown in Supplementary Figure 1.

Supplementary Figure 3 depicts pictures of the uncropped immunoblots shown in Figure 5a.

Supplementary Figure 4 depicts gMFI expression levels of CD127 and BCL2.

Supplementary Figure 5 shows that transgenic expression of BCL-xL in NCOR1 cKO^{Cd4} mice did not restore the fraction of CD69^{+} thymocytes.

Supplementary Figure 6 shows RORγt expression during positive selection in WT and NCOR1 cKO^{Cd4} thymocytes and CD55 expression in peripheral WT and NCOR1 cKO^{Cd4} CD4^{+} T cells.

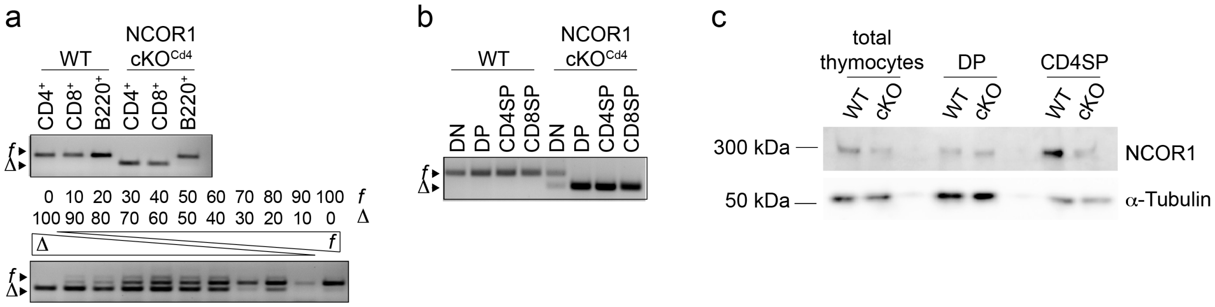

Supplementary Figure S1. Deletion efficiency of NCOR1 in NCOR1 cKO^{Cd4} T cells

(**a**) PCR analysis of DNA isolated from sorted splenic WT (*Ncor1*$^{f/f}$) and NCOR cKO^{Cd4} CD4^{+} and CD8^{+} T cells and splenic B220^{+} B cells to detect deletion of *Ncor1* (upper panel). Lower panel: DNA from sorted splenic WT (*Ncor1*$^{f/f}$) B cells and NCOR cKO^{Cd4} CD4^{+} T cells were mixed at the indicated ratio and PCR was performed to determine the sensitivity of the PCR reaction to detect the deletion of the floxed *Ncor1* allele. The sizes of the PCR fragments are either 346 bp for the floxed (*f*) or 246 bp for the deleted (Δ) allele. (**b**) PCR analysis of DNA isolated from sorted *Ncor1*$^{f/f}$ and *Ncor*1$^{f/f}$*Cd4*Cre DN, DP, CD4SP and TCRβ^{hi} CD8SP thymocytes. (**c**) Immunoblot analysis showing the expression of NCOR1 in total thymocytes and in sorted DP and CD4SP thymocytes isolated from WT and NCOR1 cKO^{Cd4} (cKO) mice. 2 x 10^{6} cells were used for each population. α-tubulin levels were used as loading control. Data in (**a**) are representative of 2 (upper panel) and 1 (lower panel) independent samples analyzed in 2 (upper panel) independent experiments or in 1 (lower panel) experiment. Data in (**b**) are representative of 1 sample (WT) or 2 (NCOR cKO^{Cd4}) independent samples analyzed in 1 (WT) experiment or 2 (NCOR cKO^{Cd4}) independent experiments. Data in (**c**) are representative of 2 independent samples analyzed in 2 different experiments for DP and CD4SP subsets and for 4 independent samples analyzed in 4 experiments for total thymocytes. (**a,b,c**) All pictures were cropped from bigger gels and blots. The uncropped pictures are shown in Supplementary Fig. S2.

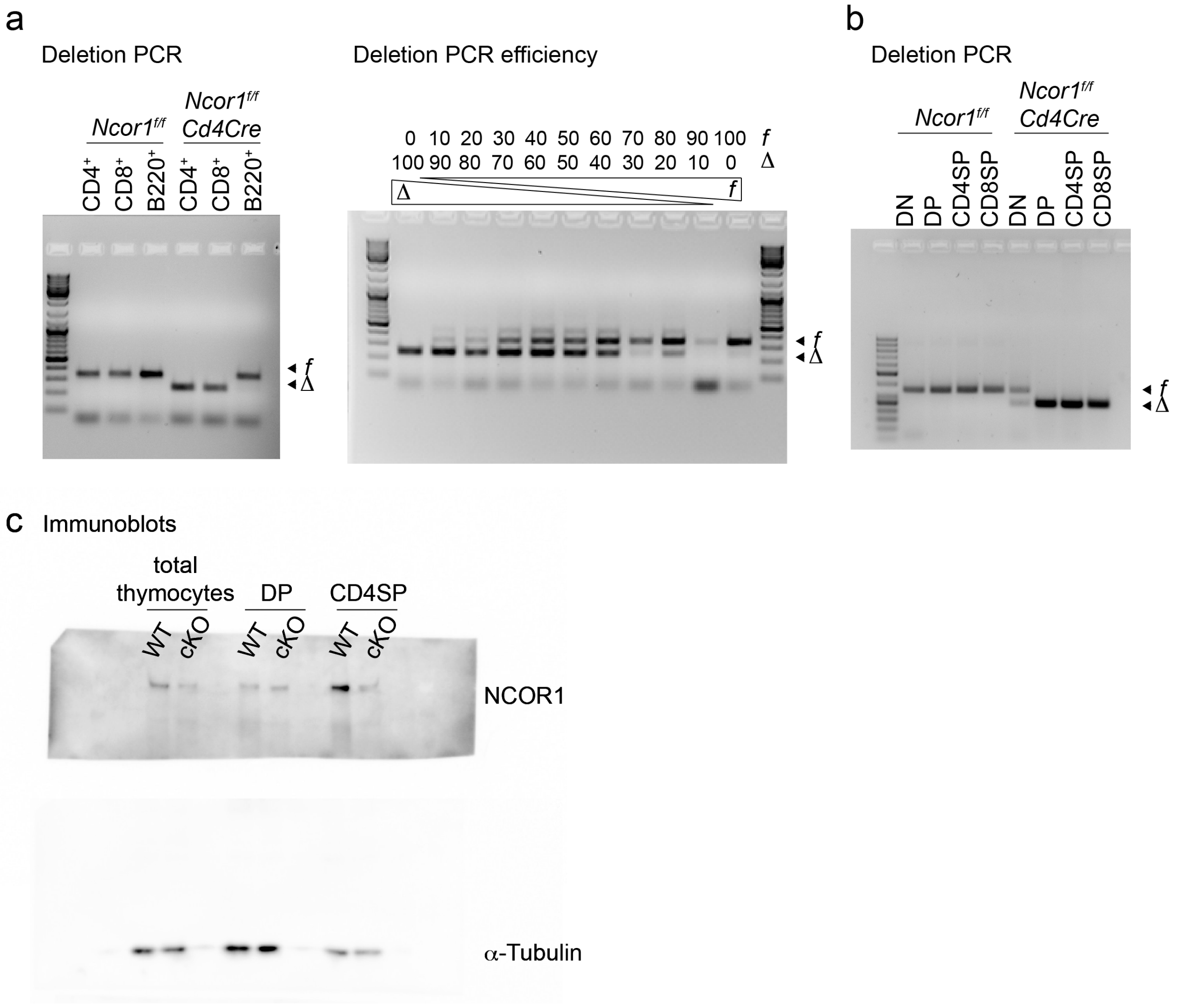

Supplementary Figure S2. Uncropped pictures for Supplementary Figure S1.

Pictures in (**a**) and (**b**) show uncropped DNA agarose gels for Supplementary Figs. 1a and 1b, respectively. Pictures in (**c**) depict uncropped immunoblots for Supplementary Fig. 1c. The DNA size markers in (**a**) are GeneRuler Ladder mix (Fermentas) and in (**b**) agarose gel 50bp DNA ladder (Fermentas).

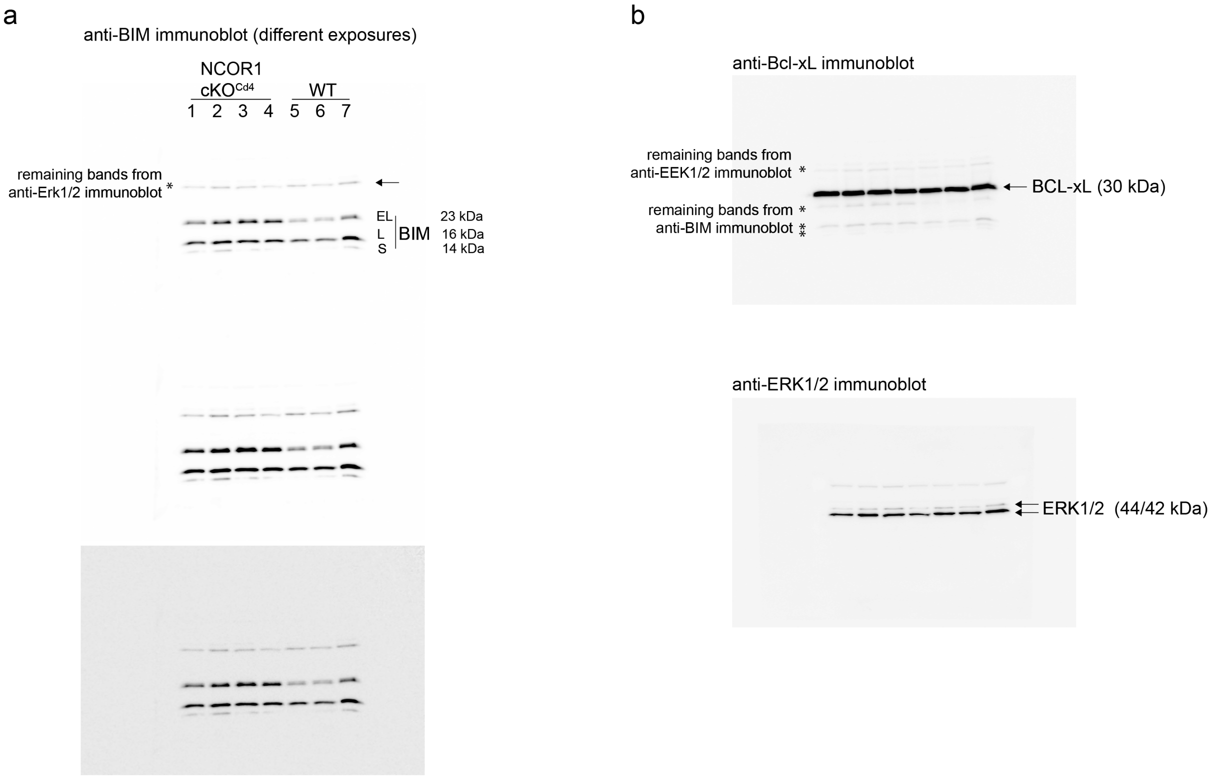

Supplementary Figure S3. Uncropped immunoblots for Figure 5a.

(**a**) Different exposures of the anti-BIM immunoblots. The three BIM isoforms (BIM_{EL}, BIM_{L}, BIM_{S}) are shown. (**b**) The upper panel depicts the anti-BCL-xL and the lower panel the anti-ERK1/2 immunoblots. (**a** and **b**) The immunoblot was first probed with the anti-ERK1/2 antibody, then with the anti-BIM antibody and finally with the anti-BCL-xL antibody.

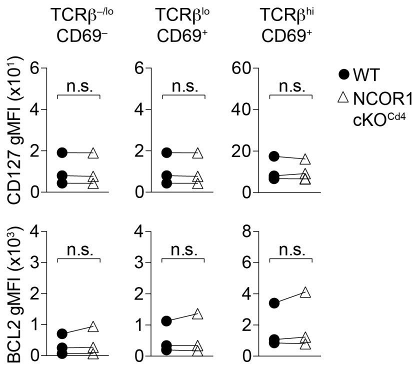

Supplementary Figure S4. CD127 and BCL2 expression are similar in TCRβ^{hi}CD69^{+} WT and NCOR1 cKO^{Cd4} DP thymocytes.

The diagrams indicate geometric mean fluorescence intensity (gMFI) of CD127 and BCL2 expression on the indicated WT and NCOR1 cKO^{Cd4} DP thymocyte subsets, and show the summary of 3 independent experiments (2-5 mice were analyzed per group in an experiment, average gMFI for each experiment is shown). The lines connect samples analyzed in the same experiment. n.s., not significant, one sample t-test (columns statistics; WT was set as 1 and relative NCOR1 cKO^{Cd4} was calculated, diagrams show absolute gMFI levels for each experiment).

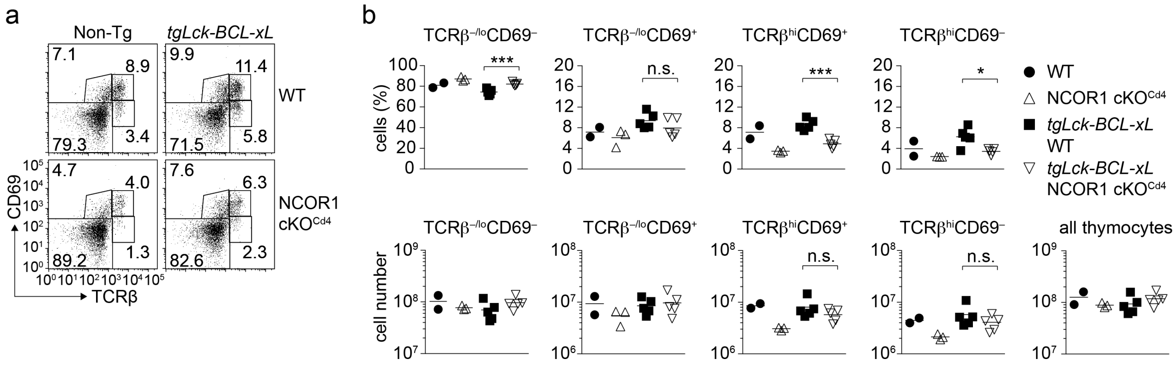

Supplementary Figure S5. Transgenic expression of BCL-xL does not rescue the generation of NCOR1 cKO^{Cd4} SP thymocytes

(**a**) Flow cytometry analysis of CD69 and TCRβ expression on thymocytes isolated from WT and NCOR1 cKO^{Cd4} mice that are non-transgenic (left panel) or transgenic for *Lck-BCL-xL* (right panel). (**b**) Percentages and cell numbers of TCRβ$^{-/lo}$CD69^{-}, TCRβ$^{-/lo}$CD69^{+}, TCRβ^{hi}CD69^{+} and TCRβ^{hi}CD69^{-} thymocytes in WT and NCOR1 cKO^{Cd4} mice that are transgenic for *Lck-BCL-xL* (right panel). ***P < 0.001, n.s., not significant, unpaired two-tailed Student's t-test. Data are representative (**a**) or show the summary (**b**) of 2-5 mice analyzed in 3 different experiments. The data for WT and NCOR1 cKO^{Cd4} mice show the littermate controls from the transgenic *Lck-BCL-xL* intercrosses.

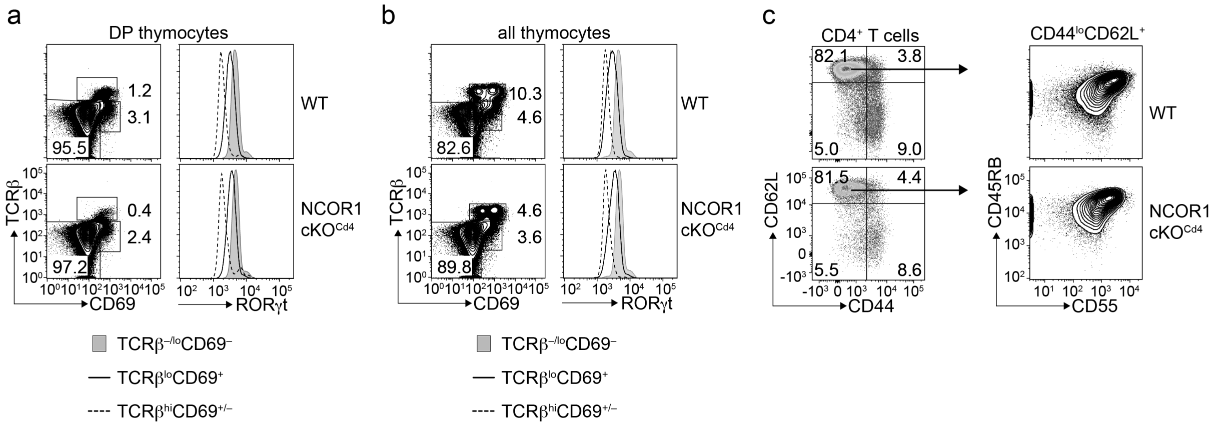

Supplementary Figure S6. Similar downregulation of RORγt expression in positively selected WT and NCOR1 cKO^{Cd4} thymocytes and similar CD55 expression in naïve WT and NCOR1 cKO^{Cd4} CD4^{+} T cells.

Flow cytometry analysis of CD69 and TCRβ expression on (**a**) DP thymocytes and on (**b**) total thymocytes isolated from WT and NCOR1 cKO^{Cd4} mice. Histograms at the right depict RORγt expression in the indicated subsets. (**c**) Flow cytometry analysis of CD62L and CD44 expression in TCRβ^{+}CD4^{+}-gated splenocytes isolated from WT and NCOR1 cKO^{Cd4} mice. Contour plot at the right shows CD45RB and CD55 expression on naïve (CD44^{lo}CD62L^{+} CD4^{+} T cells. Data are representative of 4 (**a,b**; WT), 3 (**a,b**; NCOR1 cKO^{Cd4}), 2 (**c**; WT) and 4 (**c**; NCOR1 cKO^{Cd4}) mice analyzed in 2 (**a,b**) independent experiments and in 1 **(c)** experiment.
